# *Brassica napus* Haploid and Double Haploid Production and Its Latest Applications

**DOI:** 10.3390/cimb45050282

**Published:** 2023-05-18

**Authors:** Ewa Starosta, Justyna Szwarc, Janetta Niemann, Katarzyna Szewczyk, Dorota Weigt

**Affiliations:** Department of Genetics and Plant Breeding, Poznań University of Life Sciences, Dojazd 11, 60-632 Poznań, Poland; ewa.starosta@up.poznan.pl (E.S.); justyna.szwarc@up.poznan.pl (J.S.); katarzyna.szewczyk1@up.poznan.pl (K.S.); dorota.weigt@up.poznan.pl (D.W.)

**Keywords:** androgenesis, canola, haploidy, microspore reprogramming, plant breeding, induction lines, interploidy hybridization, QTL

## Abstract

Rapeseed is one of the most important oil crops in the world. Increasing demand for oil and limited agronomic capabilities of present-day rapeseed result in the need for rapid development of new, superior cultivars. Double haploid (DH) technology is a fast and convenient approach in plant breeding as well as genetic research. *Brassica napus* is considered a model species for DH production based on microspore embryogenesis; however, the molecular mechanisms underlying microspore reprogramming are still vague. It is known that morphological changes are accompanied by gene and protein expression patterns, alongside carbohydrate and lipid metabolism. Novel, more efficient methods for DH rapeseed production have been reported. This review covers new findings and advances in *Brassica napus* DH production as well as the latest reports related to agronomically important traits in molecular studies employing the double haploid rapeseed lines.

## 1. Introduction

Rapeseed (*Brassica napus* L., AACC, 2n = 38) is one of the main oil crops in the world. It is an amphidiploid species derived from spontaneous interspecific hybridization between turnip rape (*Brassica rapa* L. syn. *Campestris*, AA, 2n = 20) and cabbage (*Brassica oleracea* L., CC, 2n = 18). The geographical center of its origin is located around the Mediterranean coastline of Southern Europe, 5000–10,000 years ago. Compared with crops such as soybean, rice, and wheat with much longer evolutions and domestication histories, rapeseed can be considered a recently domesticated crop. Two botanical varieties have been defined: *B. napus* L. var. *rapifera* (DC) Metzger and *B. napus* L. var. *oleifera* Delile [1]. The latter of the two is known for its oil content and wide application. It is grown extensively in Europe, Canada, China, India, and Australia (USDA 2022). In the nineteenth century in Europe, canola oil was highly valued for its high erucic acid content required for the lubrication of steam engines. Moreover, due to high protein levels, it was used as animal feed. In the following years, rapeseed underwent substantial phenotype changes and considerable improvement of agronomic traits, which resulted in a significant growth of yield and oil content. The refinement of good agronomical practices had a tremendous impact on the efficiency of canola crop cultivation. The launch of “double low” cultivars has significantly increased the popularity of canola oil. In 2022–2023, the world rapeseed oil consumption has increased to 31.8 million metric tons worldwide [2]. The presented progress has been made possible by the development of *Brassica napus* breeding programs all over the world. At present, researchers and plant breeders focus on various aspects of improving the yield and quality of rapeseed and widening its application.

The main research targets are to obtain cultivars with various, but controlled and stable fatty acid composition (dependent on the implementation of canola oil), to increase the protein and decrease the glucosinolate amount in seeds, and to create new cultivars resistant to biotic and abiotic stresses. Other breeding objectives include a lower content of fiber and polyphenols, enhancement of cold resistance and regeneration ability from damage caused by freezing, improvement of resistance to fungal pathogens, resilience to pests and vermin, herbicide tolerance, balancing the maturing of the seeds, as well as reducing the proneness to pod shattering and seed loss. Traits that increase the agronomic value of the cultivars are also being researched: improved use of fertilizers, robustness to soil quality changes (drought, flooding), alleviated tendency for crop lodging, rapid germination, covering of the space between rows to avoid weed infestation, and increasing the number of seeds per silique [3,4].

Through the years, various methods and protocols have been implemented in plant breeding programs. The leading strategy for new rapeseed cultivar development exploits the use of double haploid plants. Therefore, this review discusses production methods of DH rapeseed and its applications.

## 2. Double Haploid

A double haploid organism carries two sets of chromosomes and is homozygous at every locus; thus, genetic segregation will not occur [5]. Within the population of DH lines, the phenotype variability is high. Each of those lines has the potential of becoming a cultivar. Double haploids are widely used in molecular research for genetic map construction, gene localization, marker identification, and improvement of plant breeding efficiency.

Double haploid technology is one of the fundamental elements of plant breeding; it has been utilized in many studies for crop plant development. Primarily, the aim of DH production is the development of a true homozygous breeding line [6,7]. It offers several economic, logistic, and genetic benefits over conventional inbred lines. The main advantage of DH production is the reduced breeding time in comparison to conventional breeding, through backcrossing and selfing; moreover, the double haploid lines exhibit immortal behavior [8]. The former requires one to two breeding cycles to obtain a homozygous elite line, whereas the latter takes up to nine generations. DH lines are very useful for hybrid breeding and the development of mapping populations for molecular studies due to high genetic stability, no effect of dominance or heterozygosity, and short generation time.

The double haploid method relies mainly on the haploid cells—plant gametes are used to develop haploid embryos. In nature, such occurrence rarely happens; thus, DH production usually requires two major steps: haploid induction and chromosome doubling [9].

Plants are well known for their remarkable totipotency abilities. The cells of any type and development stage can switch into any other developmental program, form calluses, or convert into undifferentiated, proliferative growth when given the right conditions [10].

Haploid usually refers to the product of meiosis, which is a cell with a reduced number of chromosomes (n)—gametes containing only half of the chromosome complete set (2n). Based on cell origin, haploid plants can be described as maternal and paternal, derived from egg cells or pollen cells, and microspores, respectively [11]. Haploid individuals are tremendously useful in various kinds of research, for example, studies focused on induced mutagenesis analysis, where recessive mutations can be easily detected without the masking effect of dominance [12]. Haploids are usually smaller, less vigorous, and sterile. Therefore, production of double haploids is more desirable for practical purposes. Although haploid plants can form spontaneously, it is an extremely rare occurrence.

## 3. Haploid/Double Haploid Induction Methods

Different methods for haploid induction for various species are used, as their viability varies between genotypes. They can be classified in two main groups: in vivo (*in planta*) or in vitro methods. In vivo methods include gynogenesis and androgenesis. The former usually involves ovary or flower culture, while the latter involves anther and microspore culture, facilitating the microspore embryogenesis [13]. Haploid progeny can also be developed through wide hybridization or intraspecific hybridization. The *bulbosum* method is the basic example of haploid induction mediated by interspecific crossing of related species [14]. The latest reported techniques include the use of genetically modified inducer lines [11]. The efficiency of haploid/double haploid production varies depending on the method (Table 1.)

The first reports of microspore cultures were presented by Thomas and Wenzel in 1975 [24]. By the end of 1980s, it was established that embryos can be effectively generated through the cultures of isolated microspores in a hormone-free medium, omitting the callus phase [6]. Robert Lichter, [25] first developed a microspore-derived double haploid production protocol on NLN medium without hormone addition.

## 4. *Brassica napus* Microspore Embryogenesis

Microspore embryogenesis refers to the transition of male gametes from their natural development pathway towards embryo or callogenesis. Precursors of pollen grains in diploid plants are microspores, which usually contain a haploid number of chromosomes. Given the right conditions, it is possible to grow mature plants from microspores; however, the effectiveness of embryogenesis depends on various factors. The main variable is species or genotype, although the developmental stage of the microspores must also be considered. Some species induce immediately in early stages of microspore genesis; however, in most cases the first pollen meiosis is needed [26]. *Brassica napus* embryogenesis is most effective in a highly vacuolated microspore development stage, just before pollen mitosis [27,28].

Another important aspect to consider is the selection of the proper stressor or embryogenesis technique adapted to the donor species. The most used factors include heat, cold, starvation, chemical induction, or the combination of more than one element. Rapeseed microspore embryogenesis protocols usually use a heat stress treatment (usually 32–33 °C) [29]. Some protocols include cold treatment prior to heat manipulation, although the effectiveness of such a method may be lower in *B. napus* winter types than spring types, due to their high cold tolerance [30,31,32]. Nevertheless, Pem et al. [32] proved that extended exposure to lower temperatures (18 °C) induces embryogenesis in rapeseed and provides two different pathways of embryo formation: suspensor-like and multicellular embryos without suspensor. The use of hormone treatment in embryogenesis has also been studied and found to be effective with jasmonic acid (JA) and abscisic acid (ABA). A concentration of 0.5 mg/L and treatment time of 12 h yielded 68% plantlet regeneration with ABA, and 1.0 mg/L JA for 24 h with 54% plantlet regeneration compared to untreated microspores [33].

In *B. napus*, haploid plants are usually produced via microspore culture. Rapeseed is considered the model species for studying microspore-derived plants. Anther culture is considered decent, although a less efficient method of obtaining haploid embryos in *B. napus* [34]. Haploid production efficiency through anther culture can be tenfold lower than isolated microspore culture [17]

**Figure 1:**
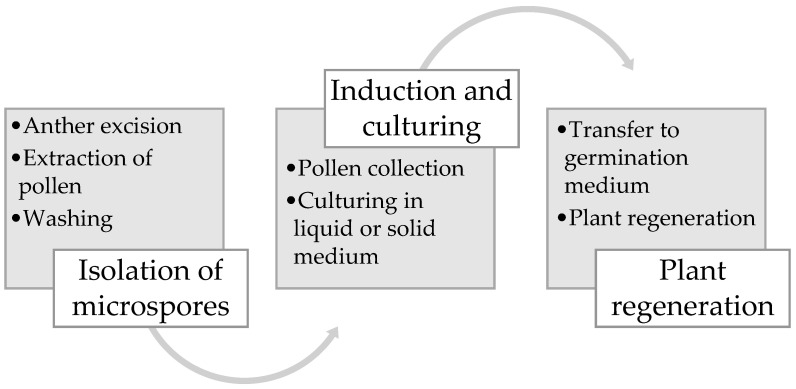
Production of microspore-derived plants.

The production of microspore-derived plants requires three main stages: isolating, culturing, and induction of the microspores; embryo selection; and plant regeneration (Figure 1).

The composition of the liquid medium used for induction must be strictly designed for the donor species, in order to deliver all nutritional components for optimal development of the haploid embryos. Solid regeneration medium, complying with the optimal osmotic pressure, makes it possible to achieve the highest plant regeneration rate possible. Routinely used plant regeneration mediums include B5 (Gamborg) medium and MS (Murashige and Skoog) medium with slight modifications, such as hormone additives and various concentrations of basal mediums (1/2 or ¼) [35,36]. The density of 10–40,000 cells per 1 mL of medium is preferable in a rapeseed microspore culture [37].

Growth factors, inhibitors, and other additives may also influence the effectiveness of haploid embryo induction. For example, metascapases (MCAs), a group of cysteine proteases, have been reported to improve in vitro embryogenesis. Downregulation of MCAs and inhibition of autophagy decreases cell death and increases embryonic development [27].

### 4.1. Mechanisms of Microspore Reprogramming to Embryogenesis in Brassica napus

**Figure 2:**
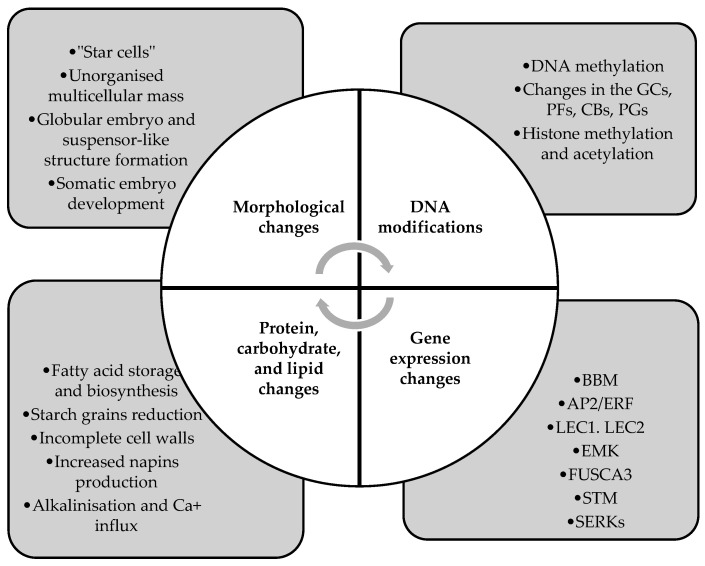
Mechanisms of microspore reprogramming in *Brassica napus*.

Even though microspore embryogenesis has been a widely utilized and studied method for haploid and double haploid production, molecular mechanisms of developmental program transition in rapeseed are still not clear. It is known that several basic metabolic and cellular processes take place during gametophytic to sporophytic transition. Morphological changes in microspores are accompanied by alterations in gene and protein expression patterns, as well as a decrease in lipid and carbohydrate content in the cells (Figure 2) [38].

#### 4.1.1. Morphological Changes

The formation of microspore-derived embryos is different from zygotic embryogenesis. Embryogenesis-induced microspores usually form round cells with centrally located nuclei and vacuoles divided by cytoplasmic strands. These cells are referred to as “star cells”, due to their morphology [39]. At later stages, an unorganized, multicellular mass that develops into globular embryos is observed. The formation of a suspensor-like structure, observed in zygotic embryogenesis, is a relatively rare occurrence in microspore embryogenesis, although it is not uncommon. The development of an embryo with a suspensor is dependent on the microspore induction and culture conditions. Usually, a suspensor develops when induction takes place at 25 °C or lower, or when an additional step of cool-down after standard heat shock treatment (32 °C) is applied. The generation of a zygote-like microspore-derived embryo is preceded by polarized microspore cell division. It has also been found that the emergence of zygotic-like embryos with suspensors is dependent on polar auxin transport [40]. Further stages of embryo development are similar to somatic embryo development with the globular, heart, and torpedo embryo developmental stages [41,42,43,44,45]. The germination of microspore-derived plants may represent a major setback in the double haploid rapeseed production, as the regeneration of plantlets varies from 5% to 30%, depending on the genotype. Furthermore, the developmental stage of the microspore-derived embryo influences regeneration efficiency. Kott and Beversdorf found that embryos transferred to the germination medium after 35 days germinated, 3-5-fold better than those transferred after 21 days. Germination rate drops significantly at 35–49 days [46]. Plant regeneration efficiency from microspore-derived embryos may also be improved via medium optimalization, chilling, desiccation, or cotyledon excision at late cotyledon stage [47,48,49].

#### 4.1.2. Protein, Carbohydrate, and Lipid Changes during Microspore Embryogenesis

The changes of fatty acid storage and biosynthesis in microspore-derived embryos are the same as in zygotic embryos [50,51,52]. Pifanelli et al. [53] determined that the SAD (stearoyl-ACP desaturase) and EAR (enoyl-ACP reductase) genes are expressed both in sporophytic and gametophytic cells; however, they are regulated differently which leads to diversified lipid patterns. Starch grains in the embryogenic cells are scarce or do not appear at all, in contrast to the cells following the microspore development program [54]. Large starch stores in the cytoplasmic area are a good indicator of the non-embryogenic development of microspores cultured in vitro [55]. Other signs of embryogenic commitment include formation of irregular and incomplete cell walls, caused by increased callose synthesis and deposition of excreted cytoplasmic material, as well as a decrease in cellulose synthesis [56]. The cell walls during embryogenesis become fragile and prone to deformation [57]. Another interesting finding suggests an autophagic character of plastids of microspore-derived embryos. Their activity is a part of a cytoplasm digestion and excretion program necessary for embryogenic switch [58].

Increased production of napins, the water-soluble storage proteins of *B. napus* accumulated in seeds, have been observed during microspore embryogenesis. Expression of these proteins is regulated by 10–16 napin-encoding genes [59]. In the 1990s and 2000s, several studies were conducted, leading to the discovery of a coincidence of napin expression upregulation and its use as a marker for detecting induction of *B. napus* microspore embryogenesis and embryo development [60,61].

Pauls et al. (2006) described in depth the role of cellular pH shift towards alkalinization, Ca^+^ influx, and its putative involvement in heat-induced microspore embryogenesis. Heat shock initiates a cascade of reactions in the microspore, altering ROPs’ (Rho of plants) gene expression, and resulting in cellular morphogenesis and embryo development. Solis et al. [62] explored the behavior of PMEs (pectin methylesterases), that is, methyltransferases that take part in cell wall remodeling. Even though the low expression of *BnPME* genes has been previously associated with microspore embryogenesis, the results demonstrated the expression patterns comparable to zygotic embryogenesis. Stress-induced microspore embryogenesis increases auxin production as well as TAA1 (tryptophan aminotransferase) and NIT2 (nitrilase) genes’ expression of auxin biosynthetic pathways and progresses further during embryo development. Furthermore, it was stated that polar auxin transport (PAT) inhibits embryogenesis [63].

#### 4.1.3. DNA Modifications

Stress-induced microspore embryogenesis can result in DNA structure changes. Such modifications cause a shift in the nuclear architecture during plant differentiation and proliferation. Global DNA methylation decreases with the epigenetic reprogramming after embryogenesis induction [64]. Heat shock (32 °C) applied to *B. napus* microspores no longer than 6h leads to DNA hypomethylation. An approximately twofold change was observed [65]. Segui-Simarro et al. [66] noticed changes in size, shape, and distribution of interchromatin structures such as granule clusters GCs, perichromatin fibrils PFs, Cajal bodies CBs, and perichromatic clusters PGs during the transition of the microspore development program from gametophytic towards embryogenic. The authors suggested that remodeling in the interchromatin domain may be the cause of transcriptional changes. Consequently, RNA-associated structures can be a regulatory mechanism in microspore embryogenesis. In addition, changes in histone methylation and acetylation have been found. Microspore reprogramming is followed by spatial and temporal alterations in distribution patterns of methylated H3K9m2 and acetylated H3/H4 histones, which are equal to *BnHKMT* and *BnHAT* genes’ (methyltransferase and acetyltransferase, respectively) expression patterns. The authors support the hypothesis of histone acetylation and methylation taking part in embryogenic reprogramming of the microspores [28]. Further endorsement of this statement provides a standalone analysis of histone deacetylases (HDACs) and its role in regulating haploid embryogenesis. Blocking HDAC activity with trichostatin A resulted in hyperacetylation of histones H3/H4 and a high yield of microspore-derived embryos [67].

#### 4.1.4. Gene Expression Changes

Gene expression studies of early microspore embryogenesis have been previously conducted, and this section summarizes the most significant findings. Transcriptional changes caused by embryogenesis induction involve stress adaptation genes, pollen development, and embryogenic transition. A majority of differentially expressed genes have been described as stress adaptation genes, but they do not contribute to microspore embryogenesis. One of the widely researched genes associated with embryogenic development is the BABY BOOM gene (BBM). At the beginning of the 2000s, BABY BOOM genes captured scientists’ attention and have since been extensively studied [68,69,70,71]. BBM genes are the transcription factors of the AP2/ERF family (APETALA2 FAMILY/ETHYLENE-RESPONSIVE ELEMENT BINDING FACTOR). Its broad spectrum of functions includes cell proliferation, plant growth, and development regulation [69]. BBM genes have been reported to participate in microspore reprogramming to embryogenesis. However, its exact role in *B. napus* induction of microspore embryogenesis and development of microspore-derived embryos is not clear. Boutilier et al. [70] found that BBM genes express mainly in developing embryos and seeds. BBM promotes cell proliferation and morphogenesis, though its embryogenesis-inducing nature is not yet identified. On the contrary, analysis of expression of BBM-targeted genes in *Arabidopsis thaliana* showed large groups of genes expressed in meristems, responsible for actin activation, cell differentiation, proliferation, and cell wall modifications. It was concluded that BBM genes activate developmental pathways leading to plant growth; therefore, they may be the starting point of microspore redifferentiation [71]. The LEAFY COTYLEDON genes (LEC1 and LEC2), EMBRYOMAKER (EMK), FUSCA3, and SHOOTMERISTEMLESS (STM) seem to be related to microspore-derived embryo development; however, they have not been correlated with the induction of microspore embryogenesis [29,72,73,74,75,76]. Other genes discovered through analysis of differentially expressed genes (DEGs) have been reported to carry unknown function and have never been connected with microspore embryogenesis [77]. Later, the DEGs were subtracted using the suppression subtractive hybridization (SSH) method. A total of 254 ESTs were isolated, of which 96.4% were homologous to known genes: 42.7% of unclassified proteins and 13.6% of metabolism genes. Six genes were examined using qRT-PCR, and their expression was confirmed in different stages of embryogenesis [78]. One of the gene groups that have been found participating in embryogenesis is the SERK (somatic embryogenesis-related kinase) family. These genes encode leucine-rich repeats transmembrane receptor-like proteins (LRR-RLK). SERKs are part of a brassinosteroid receptor complex involved in brassinosteroid signaling. Increased expression level has been observed in BnSERK1 and BnSERK2 genes during microspore culture and development [79].

## 5. Chromosome Doubling

In addition to homozygosity, the microspore-derived haploid plants ensure a wide spectrum of genetic recombination. As previously mentioned, they may display reduced vigor and infertility due to the meiotic division failure.

Some of the haploid individuals undergo spontaneous doubling. Generally, to obtain a relatively high percentage of homozygous fertile plants from microspore-derived plants, various chemical compounds are used. Induced doubling of chromosomes can be conducted both in vivo and in vitro. The most frequently used chromosome doubling agent is colchicine, which disrupts normal mitotic cell division. Colchicine can be used on isolated microspores, microspore-derived embryos, and regenerated plants [5,18,19]. The use of colchicine results in 16% to 94% of individuals with doubled chromosome numbers in in vitro culture. Microspore treatment can have a greater chromosome doubling effect compared to microspore-derived embryo treatment or regenerated plant treatment, which makes it possible to omit the haploidization stage of double haploid production [80,81,82]. Other antimitotic agents such as oryzalin, trifluralin, and amiprophos-methyl can also support chromosome doubling [83,84]. Other protocols implement the use of heat shock to induce the doubling of chromosomes.

Even though microspore embryogenesis and colchicine treatment for double haploid production is a quite efficient method, it is also time- and cost-consuming and requires laboratory expertise. *B. napus* is a naturally derived hybrid obtained by crossing *Brassica rapa* and *Brassica oleracea*, followed by chromosome doubling That is why it may serve as a model species for genome duplication research. Some plant species can yield haploid embryos through crossing of specific individuals carrying particular genetic traits [85].

## 6. *Brassica napus* Haploid Production via Interploidy Hybridization

In *B. napus*, the first study regarding the doubled haploid production via interploidy hybridization was reported in 2018 [22]. A *Brassica* allooctoploid pollen donor (AAAACCCC, 2n = 8x = 76) acted as a doubled haploid inducer of *B. napus*. The described method can provide homozygous lines after one generation without the application of colchicine treatment. The genetic composition of the obtained DH in most cases resembles the maternal chromosome set, with the elimination of paternal genome. It should be noted that the stability of the DH induction is genotype-dependent. The number of obtained double haploids is highly variable and spans between 34.09–98.66% [86]. The induction efficiency of DH inducers is dependent on the karyogene and cytoplasmic genotype. Thus, it was concluded that the induction effect is affected by the interaction between the maternal karyogene and cytoplasmic genotype [86]. Lou et al. [87] found a specific insertion on chromosome C03 in the induced DH individuals. It may indicate that the insertion of the paternal chromosome is not a random occurrence. The exact mechanism of DH induction with the use of *Brassica* allooctoploid as a pollen donor is not known; however, it is assumed that aneuploidy is one of the factors contributing to the successful DH induction in *B. napus*. Location and cloning of the genes regulating inducibility will become the priority for the studies in the near future, as it is one of the most promising and efficient in vivo methods for chromosome doubling in *B. napus*.

Despite the ambiguity and novelty of this technique, it has by now found an implementation in scientific studies. Zhang et al. [88] developed cytoplasmic male sterile and maintainer lines of *B. napus* with the doubled haploid inducer allooctoploid lines Y3380 and Y3560 described above. Zhou et al. [89] hypothesized that the new DH inducer lines can successfully replace the method of continuous backcrossing of interspecific F_1_ with the parent to resolve the problem of low seed rate. Such difficulty is caused by hybrid sterility and chromosomal mismatch in the hybrid offspring of rapeseed. In the study, it was found that the mother egg cell and egg cell genetic stability were significantly higher than that of a sperm cell. This research provides valuable data that may contribute to reducing the time of novel rapeseed germplasm development.

## 7. *Brassica napus* Haploid Induction Lines

Haploid induction lines (HI) have been used and researched mainly in monocotyledon crops such as maize and rice with great success. It is an in vivo method of obtaining haploid plants within one generation. The technique is based on the use of haploid inducer lines as pollen donors. The result of such pollination is the stimulation of haploid eggs to produce embryos with the loss of parental chromosomes. Such an outcome is the result of a loss-of-function mutation of DMP (DOMAIN OF UNKNOWN FUNCTION 679 membrane protein) genes. Maternal HI systems have progressed rapidly with the identification of HI genes (*Zea mays*) [20], although they have been found mainly in monocotyledons. The lack of such findings in dicotyledon plants is a great disadvantage in breeding development.

Zhong et al. [21] confirmed that the DMP loss-off-function mutation induced the in vivo maternal haploid production in several dicot plants. DMP-HI system has been found effective in dicot plants such as *Arabidopsis thaliana*, *Medicago truncatula*, *Solanum lycopersicon*, *Solanum tuberosum* L., *Brassica napus*, and *Nicotiana tabacum* [90,91,92,93]. The DMP-HI system is genotype-independent. DMP genes can be easily selected through their sequence identity and expression in generative organs or cells. In *Brassica napus*, DMP genes have been determined through a search of maize ortholog genes expressed in flowers and flower buds. Researchers suggest that targeting *BnaDMP* genes can be used for haploid production in *Brassica napus*. Li et al. [94] developed haploid-induced lines through knockout of DMP (Domain of unknown function 679 membrane protein). Zhong et al. [21] constructed a pipeline for the establishment of DMP orthologs and proved the functionality of *dmp*-based maternal HI (haploid induction) system in *B. napus*. In vivo HI technique was established for the mutation of DMP genes. Authors found that the amphihaploid induction rates are 1.1% and 2.4% while crossing double and triple mutants, respectively. The *dmp* mutation in one *B. napus* genotype can be used to induce amphihaploids in various maternal genotypes. It is one of the latest perspectives of in vivo haploid induction through mutation of DMP maternal haploid inducer genes [93]. In both studies, CRISPR/Cas9 mutagenesis constructs designed for knock-out mutation induction were used, facilitating fluorescent protein markers for easy identification of mutants. It was concluded that mutation of *B. napus* DMP genes contribute to in vivo haploid embryo development upon selfing in various genotypes.

## 8. Recent Findings Contributing to *Brassica napus* Double Haploid Plants

Double haploid rapeseed may be implemented in various molecular studies. DH provides genetic variability, stability, uniqueness, and shortened development time at low cost. The original purpose of DH technology was to rapidly produce homozygous individuals with fixed genetic composition. Nowadays, their advantages are used to conduct many new research studies leading to new findings [95,96,97,98,99]. They are the foundation of genetic and linkage mapping, localization of new traits and molecular markers, as well as quantitative trait locus (QTL) analysis and marker-assisted selection (MAS). In the last five years, studies using double haploid *B. napus* has provided great insight into genes/QTLs (Table 2) and molecular mechanisms behind agronomically important traits. To prove the value and applicability of DH technology in rapeseed, this section will focus on the latest findings with DH *B. napus* used as the research material.

### 8.1. Plant Structure

Plant height, branching, silique number, and number of seeds in siliques are important rapeseed agronomic traits affecting the yield. High-stemmed plants are more prone to lodging caused by strong winds and decrease the efficiency of the harvesting machinery. Higher branching rate in the plants results in a higher yield of a single plant. The number of harvested seeds directly reflects the agronomic and economic value of a cultivar. In the last five years, several studies have been conducted using *B. napus* double haploid lines and the bulk segregant analysis approach, their main objectives being the identification of genes or QTLs conferring the traits described above. Bulk segregant analysis is one of the several SNP (single nucleotide polymorphism) identification strategies, such as GWAS (genome wide association study), ESTs (expressed sequence tags), array-based analyses, amplicon sequencing, or identification from sequenced genomes [122,123,124]. It is a method based on the use of segregating populations of two bulked pools of individuals with contrasting traits. Supplemented by next-generation sequencing (NGS) of the whole genome or transcriptome, this approach allows identification of molecular markers linked to a gene of interest, manifesting itself in the phenotype in a rapid and relatively low-cost and non-time-consuming way.

Zhao et al. [125] and Wei et al. [126] provided new insights regarding the molecular mechanisms underlying dwarfism of *B. napus*. Using the “Aiyuan1” DH lines and “Zhongyou 821” cultivar, a new dwarf locus—DS-4 encoding nucleus-targeted auxin/indole-3-acetic acid (Aux/IAA) protein—was identified. The P87L substitution in the highly conserved region of Aux/IAA was found to cause extreme dwarfism. Similarly, Wei et al. (2021) found a ndf-2 mutation of Aux/IAA on chromosome A03. Both affect the auxin signaling pathway. Additionally, nine height-related candidate genes were found on chromosome A03 (*BnaA03g31770D*, *BnaA03g37960D*, *BnaA03g24740D*, *BnaA03g40550D*, *BnaA03g26120D*, *BnaA03g35130D*, *BnaA03g42350D*, *BnaA03g25610D*, and *BnaA03g39850D)* that are involved in gibberellin and cytokinin signaling pathways [127]. In addition to the IAA content and signaling pathways, the modification changes in the ABA and GA (gibberellin) biosynthesis were found [128].

### 8.2. Number of Inflorescence, Seeds, and Pod-Shattering Tendencies

The number of seeds per silique is another trait that reflects the agronomic value of the rapeseed. In the study, the BSA (bulked segregant analysis) method was conducted in combination with *Brassica* 60K Illumina SNP array. The rapeseed double haploid lines were derived from a 6Q006 × 6W26 hybrid. A major QTL region on chromosome A09 was found. Further BSA-seq analysis detected an InDel variation of BnaC09g45400D gene encoding adenine phosphoribosyltransferase 5 (APT5) [129]. Pod length is a fundamental factor affecting seed yield, as longer pods usually mean a higher number and weight of seeds. Pod length is reported to be a multigenic controlled trait. Markers at two loci were found using BSA and random amplified polymorphic DNA with rapeseed double haploid line “Quantum” (long pod) and “China” (short pod). The two selected markers effectively identified DH lines with average pod length increased by 15% [130].

Pod-shattering in *B. napus* is a major issue, causing a considerable yield loss. Cultivars with low pod/silique-shattering make it possible to save a significant part of the yield and ensure more profitable crop farming with the use of heavy machines and highly automated agriculture. Chu et al. [131] identified a lignified-layer bridge (LBB) exclusive to an elite line OR88 and found that LBB structure is controlled by a single recessive gene. The proposed candidate gene BnTCP.C09 was found to be highly downregulated in the aforementioned line.

### 8.3. Flowering Time

In the years 2017–2023, several research articles provided new insights into genetic factors involving the flowering time (FT) of *B. napus*. Recognizing and controlling the flowering time in the available registered cultivars allows for the selection of the appropriate cultivar for the local cropping system. A total of 306 genes involved in the control of flowering time in *Arabidopsis thaliana* have been reported [132]. As *A. thaliana* is a close relative of *B. napus*, the identification of FT genes in rapeseed is considerably easier. *BnFLC.A2*, *BnFLC.C2*, and *BnFLC.A3b* genes are major factors determining FT in *B. napus* and are reported to have an additive effect [133]. Major QTLs on chromosome C1 and C9 were also reported to have additive characteristics [134]. Thirty-six genes associated with flowering time were identified through whole-transcriptome analysis of double haploid semi-wintertime line “Ningyou7” [135]. One QTL of the *B. napus* introgression line was found to carry an allele introgressed from *Brassica oleracea* that affects the flowering time [136]. Two QTLs were detected on chromosome A02 of *B. napus*. One of the two QTLs contained a *BnaFT.A2* flowering gene [137].

### 8.4. Seed Traits

The yellow seed trait is a widely researched topic in *Brassica napus* and other *Brassica* oilseed crops. Yellow seeds are associated with high oil, low fiber, and pigment content [138]. Molecular mechanisms controlling the yellow color are the center of attention for many researchers and breeders. The KNDH double haploid line was derived from progeny of yellow-seeded *B. napus* N53-2 with high oil content and black-seeded Ken-C8 with low oil content. Ten QTLs were detected, out of which four were stable in multiple environments. The QTL *cqSC-A09* was found to control the pigmentation of the seeds as well as oil and fiber content [139]. A high-density linkage map was constructed with the double haploid population KNDH. Identified QTLs were associated with SOC or SPC (seed oil content or seed protein content) [138]. Another two double haploid lines were developed from black-seeded cultivars’ progeny and tested in various environments. Segregating bulks were revealed based on the distribution of lignin (black pigment) in the seeds. Low lignin content in the seed coat is correlated with high oil content [140]. SNP analysis indicated a narrow genomic region for low lignin content [141].

Seed weight is another indicator of the agronomic value of oilseed canola. BSA-based analysis revealed four genes: *GSBRNA2T00037136001*, *GSBRNA2T00037157001*, *GSBRNA2T00037129001*, and *GSBRNA2T00069389001* being the putative candidate genes for seed weight control [142]. Major QTL *cqSW.A03-2* explains the large percentage of phenotypic variations in analyzed *B. napus* double haploid lines. The candidate gene of said QTL locus may be the *BnaA03G37960D* gene [143] Major QTL mapping conducted by Zhang et al. [144] confirmed the phenotypic effect of *qSW.C9* QTL influencing seed weight and observed its mediating character toward proliferation, cell expansion, and signaling pathways.

### 8.5. Resistance to Diseases

#### 8.5.1. Sclerotinia Stem Rot

Genetically based resistance to *Sclerotinia sclerotinium* is poorly understood in crops. It is known that the resistance is controlled by several genes, which restrict the development of resistant cultivars. Hence, the need for identification of QTL and molecular markers is a pressing problem, and the development of segregating populations and genetic maps is vital. Behla et al. [111] carried out a quantitative trait loci analysis in three rapeseed double haploid populations. Three to six QTLs were found in each population; common QTLs were identified in linkage groups A7, C6, and A9. A comparative transcriptomic study revealed 36 upregulated, putative candidate genes responsible for ST resistance [145]. In addition, Wu et al. [112] stated that ST resistance correlates negatively with flowering time. Four QTLs colocalize for stem rot and early flowering time, which reveals a genetic link between the two traits.

#### 8.5.2. Clubroot

*Plasmodiophora brassicae* Woronin, the causal agent of clubroot disease, infects the roots of *Brassica* host plants, resulting in characteristic clubs. These formations hinder water and nutrient flow through the roots, and consequently the plant. Clubroot resistance (CR) genes are being continuously researched. Up to today, over twenty genes and QTLs have been identified. In recent years, several researchers have contributed to this number. CR genes and genes taking part in the plant’s defense response, as well as new molecular markers, have been found in *B. napus* [106,109,146,147]. Shaikh et al. [148] studied the inheritance of resistance of three *Plasmodiophora brassicacae* pathotypes using two rapeseed double haploid lines. Four percent of the DH lines were resistant to all studied pathotypes, which led to the conclusion that pyramiding several resistance genes is a promising approach to clubroot-resistant rapeseed breeding. Double haploid *B. napus* lines were also proven useful in transcriptomic and metabolomic approaches, providing valuable information on early-stage responses to clubroot in resistant and susceptible *B. napus* lines, as changes in organic acid, amino acids, sugars, mannitol accumulation in roots, pyrroline-5-carboxylate, citrate synthase, and pyruvate kinase expression were observed [148].

#### 8.5.3. Blackleg

*Leptosphaeria* spp. causing blackleg disease in *Brassica* species is a major threat in rapeseed breeding. The greatest yield loss is caused by the *Leptosphaeria maculans* species. The annual yield loss in Poland in rapeseed crops is reported to be approximately 10–60%, depending on the region [149]. Worldwide, some disastrous cases of 90% yield loss were recorded in France and Canada [150,151]. The strategies of blackleg disease control include cultural practices such as crop rotation, soil tillage, fungicides, and chemical agents. However, such solutions are neither sufficient nor economically feasible. The implementation of resistant cultivars using the major, race-specific resistance genes (R genes) in present agriculture systems seems to be the best blackleg management method. However, strong selection pressure created by large-scale monoculture rapeseed plantations put on the local strains of *Leptosphaeria maculans* strains results in relatively fast breaking of R genes. The resistance of several commercial cultivars was reported to collapse within three years after their release [152]. The pathogen ability of rapid evolution is possible because of its resilience and the fact that it can reproduce both sexually (ascospores) and asexually (pycnidiospores). Quantitative resistance (QR) originating from minor, non-race specific resistance genes is said to be a more stable source of resistance in rapeseed in terms of long-term viability. The QR-controlling mechanisms are still unclear, although they are considered the most desired and durable rapeseed protection mechanisms. Quantitative trait loci (QTL) analysis is the main method for studying QR, especially with rapidly developing sequencing and computing techniques. Modern molecular methods significantly accelerate the identification of blackleg resistance genes, both major and minor, and molecular marker development. Most experiments in QTL mapping utilize the double haploid (DH) populations. Primarily, the marker system of choice for the map construction required for mining QTLs conferring blackleg resistance in *B. napus* has been SSRs; however, the SRAP, AFLP, RAPD, RFLP, and SCAR markers have been also utilized [153,154,155,156,157,158]. Raman et al. conducted several studies regarding blackleg resistance in *B. napus*. In 2018, genomic regions involved in quantitative resistance were studied in various environments—shade house and field conditions. The plant material consisted of 258–276 double haploid lines derived from Darmor-*bzh* × Yudal cross. Twenty-seven QTLs were found on twelve chromosomes, out of which only seven were detected in more than one experiment. It was also concluded that plant height and maturity may have an impact on quantitative resistance in testing conditions [101]. As most QR effects are highly influenced by environmental conditions (genotype by environment), such a statement is understandable. Genotype by environment interaction makes identification of stable QTLs conferring blackleg resistance to rapeseed challenging. In the following study, the authors mapped both qualitative and quantitative loci for *Leptosphaeria maculans* resistance. Based on SSR, SRAP, STS, and EST-SSR markers, the R and QR loci for blackleg resistance were localized. Eleven QTL were identified, out of which only two were detected in all three field experiments [105]. Another paper by Raman et al. [159] reported identification of two marker associations for two R genes and twenty-one for QR loci. Three of them were repeatedly present on chromosomes A03, A07, and C04 in the shade house and field experiments. Huaeng et al. [102] observed a significant correlation between the growth of lesions caused by *Leptosphaeria maculans* and the resistance of plants in field experiments. The QTL investigation resulted in the identification of overlapping loci on chromosome A02 in both controlled and field conditions. Over the years, the identification of QTLs conferring blackleg resistance has been widely explored, although the complexity of GxE interaction hinders the recognition of the QR effects, control mechanisms, and, as a consequence, a low number of effective and functional markers [160].

## 9. Conclusions and Future Perspectives

Double haploid technology has undoubtedly had a great impact on rapeseed research and breeding. The development of DH lines is the main backbone of *B. napus* cultivar development. The remarkable features of these lines are readily applied in genetic and molecular studies. The superiority of DH lines over conventional inbred lines is unquestionable. However, the demand of modern agriculture for improved cultivars is greater than the breeders’ ability to meet those needs, even given such tools as double haploidy. The effectiveness and production rate of haploid and double haploid plants is still relatively low. Production of microspore-derived haploid or double haploid plants at an acceptable level requires skill, experience, and a good understanding of the species or cultivar of choice. Clearly, the understanding of the molecular mechanisms underlying embryogenesis of microspores needs to be improved. Moreover, it would be beneficial to focus on the epigenetic aspects of microspore embryogenesis. Currently, microspore culture still proves to be the most efficient and available method for the production of haploids and double haploids in *B. napus* for most laboratories. The latest reported techniques exploiting interploidy hybridization or inducer lines provide a more than satisfactory efficiency rate of double haploid rapeseed production. However, the method facilitating the inducer lines is unavailable for numerous laboratories, as it requires genetic modification. The integration of biotechnological tools with the DH technique has proved fruitful, and what is more, breeding efficiency and genetic improvement has been enhanced and accelerated.

## Figures and Tables

**Table 1:** Comparison of *Brassica napus* haploid and double haploid production methods.

	In Vitro		Semi-In Vitro/In Vivo		In Vivo
	Gynogenesis	Androgenesis	Haploid inducer lines	Interploidy hybridization	Intraspecific hybridization Interspecific hybridization
	Flower buds Placenta Ovule Ovary	Anther Microspore	-	-	Spontaneous occurrence
Yield	Haploid/Double haploid	Haploid/Double haploid	Amphihaploid	Double haploid	Haploid/Double haploid
Success rate in *Brassica napus* H/DH production	Low	Low/Medium	High	High	Low
Genotype dependent	Yes	Yes	No	Yes	Yes
Reliant on pathway conversion ability (saprophytic → sporophytic)	Yes	Yes	No	No	No
Chromosome elimination	No	No	Yes	Yes	Yes
Dependent on environmental conditions (i.e., medium composition, light, temperature, humidity)	Yes	Yes	No	Yes	Yes
Pretreatment	Yes (temperature, chemical pretreatment, irradiation etc.)	Yes (temperature, chemical pretreatment, irradiation etc.)	Yes (genetic modification)	No	No
References	[15,16]	[15,16,17,18,19]	[20,21]	[22]	[23]

**Table 2:** Latest QTL mining studies for disease resistance, seed traits, and plant architecture utilizing double haploid rapeseed.

Rapeseed Population	Trait	Experimental Conditions	Used Marker Type	Number of Genes/QTLs	Author
Darmor-*bzh* × Yudal Darmor × Samourai	Blackleg resistance	Field	SNP	16 4	[100]
Darmor-*bzh* × Yudal	Blackleg resistance	Field and greenhouse	DArT	27	[101]
Darmor-*bzh* × Yudal	Blackleg resistance	Greenhouse	SNP	8	[102]
Skipton × AgSpectrum	Blackleg resistance	Field and greenhouse	SSR, SRAP, SCAR	8	[103]
Topas × AGCastle Topas × AVSapphire	Blackleg resistance	Field	SSR, DArT	22 21	[104]
RP04 × Ag-Outback	Blackleg resistance	Field and greenhouse	DArT	21	[105]
ECD01 (*B. rapa*) × DH16516 (*B. napus*)	Clubroot	Greenhouse	SNP,	2	[106]
1CA1446.476-A1296 × Hi-Q A04-73NA × Hi-Q	Clubroot	Greenhouse	SNP	2	[107]
T19 × ACDC	Clubroot	Greenhouse	SNP	3	[108]
09CR500 × 09CR501	Clubroot	Greenhouse	SNP, SSR	2	[109]
Mendel × A07-26NR	Clubroot	Greenhouse	SSR,	5	[110]
Zhongyou 821 × DHBao604 (H1), Zhongyou 821 × DH6576 (H2), Zhongyou 821 × Westar	Sclerotinia stem rot	Greenhouse	SNP	H1: 4–6 H2: 3–6 H3: 2–6	[111]
ZP1 × D12 (*B. napus* inberd lines)	Sclerotinia stem rot	Field	SNP	4	[112]
Huashuang 5 × J7005	Sclerotinia stem rot	Field	SSR	13	[113]
Bing 409 × Zhongshuang 8	Flowering time	Field	SNP	5	[114]
IMC106RR × Wichita	Root morphology Flowering time Drought resistance	Field and greenhouse	SNP	20	[115]
Polo × Topas	Flowering time Fatty acid profile Oil content	Field	SSR	14 131 14	[116]
KenC-8 × N53-2	Flowering time	Field	SNP	55 (12 environment-stable, 43 environment-specific)	[117]
KenC-8 × N53-2	Multi-main stem trait	Field	SNP, SSR, STS, SRAP, IFLP	43	[118]
SGDH284 × 158A (derived from Sollux and Zhoungyou9988 rapeseed cultivars)	Flowering time	Field	SNP	56	[119]
Low SD line No. 935 and high SD line No. 3641	Seed density per silique	Field	SNP	28	[120]
GH06 × P174 (late flowering × early flowering	Flowering time	Field	SNP	27	[121]

## Data Availability

Not applicable.
